# Can patient decision aids help people make good decisions about participating in clinical trials? A study protocol

**DOI:** 10.1186/1748-5908-3-38

**Published:** 2008-07-23

**Authors:** Jamie C Brehaut, Alison Lott, Dean A Fergusson, Kaveh G Shojania, Jonathan Kimmelman, Raphael Saginur

**Affiliations:** 1 Ottawa Health Research Institute, Ottawa Hospital, Civic Campus, 1053 Carling Avenue, Ottawa, Ontario, K1Y 4E9, Canada; 2 Department of Epidemiology and Community Medicine, Faculty of Medicine, University of Ottawa, 451 Smyth Road, Ottawa, Ontario, K1H 8M5, Canada; 3 Research Ethics Board, The Ottawa Hospital, Civic Campus, Civic Parkdale Clinic, Suite 470, 737 Parkdale Avenue, Ottawa, Ontario, K1Y 1J8, Canada; 4 Biomedical Ethics Unit, McGill University, 3647 Peel Street, Montreal, Quebec, H3A 1X1, Canada; 5 Canadian Institutes of Health Research (CIHR), 160 Elgin Street, Address Locator 4809A Ottawa, Ontario, K1A 0W9, Canada; 6 Sunnybrook Health Sciences Centre, Rm. D470, 2075, Bayview Ave., Toronto, Ontario, M4N 3M5, Canada

## Abstract

**Background:**

Evidence shows that the standard process for obtaining informed consent in clinical trials can be inadequate, with study participants frequently not understanding even basic information fundamental to giving informed consent. Patient decision aids are effective decision support tools originally designed to help patients make difficult treatment or screening decisions. We propose that incorporating decision aids into the informed consent process will improve the extent to which participants make decisions that are informed and consistent with their preferences. A mixed methods study will test this proposal.

**Methods:**

Phase one of this project will involve assessment of a stratified random sample of 50 consent documents from recently completed investigator-initiated clinical trials, according to existing standards for supporting good decision making. Phase two will involve interviews of a purposive sample of 50 trial participants (10 participants from each of five different clinical areas) about their experience of the informed consent process, and how it could be improved. In phase three, we will convert consent forms for two completed clinical trials into decision aids and pilot test these new tools using a user-centered design approach, an iterative development process commonly employed in computer usability literature. In phase four, we will conduct a pilot observational study comparing the new tools to standard consent forms, with potential recruits to two hypothetical clinical trials. Outcomes will include knowledge of key aspects of the decision, knowledge of the probabilities of different outcomes, decisional conflict, the hypothetical participation decision, and qualitative impressions of the experience.

**Discussion:**

This work will provide initial evidence about whether a patient decision aid can improve the informed consent process. The larger goal of this work is to examine whether study recruitment can be improved from (barely) informed consent based on disclosure-oriented documents, towards a process of high-quality participant decision-making.

## Background

Informed consent is a cornerstone of ethical healthcare research, and is a required component of virtually all clinical studies conducted in modern institutions. The basic principles of informed consent were first documented as the Nuremberg Code [1] in response to Nazi war crimes. Later, these principles were refined and expanded as part of the World Medical Association's Declaration of Helsinki [2] in 1964, and its subsequent updates [3]. These fundamental documents, as well as substantial philosophical, clinical, legal, and regulatory debate [4,5] have led to a general consensus regarding key criteria for informed consent, which include: the decision to participate in clinical research must be made voluntarily and free from coercion; the decision-maker must be competent to make the decision; full disclosure of relevant information must be given; and the relevant information must be understood by the decision-maker [4].

Recent work has identified a tension between the latter two core criteria [6-8]. On the one hand, researchers, institutions, and industry sponsors seek to disclose all potentially relevant information and to ensure that legal disclosure requirements are clearly met. Such disclosure is being implemented with increasingly long and complicated patient materials [7]. On the other hand, there is increasing evidence that the existing consent process often leads to poor participant understanding. Examples abound of participants not understanding even the most basic components of the studies in which they are involved [9-22], including participants not understanding that they had been randomly assigned to treatment [23], and participants believing that their treatment was already proven effective [24]. To an extent, pressures to disclose and to aid understanding can be opposed; to date, it appears that pressures towards disclosure have been stronger than those towards ensuring participant understanding [25,26].

While the practice of informed consent has emphasized disclosure and increasing complexity, there is considerable literature on how to improve knowledge and understanding when making tough health care decisions. It is well known, for example, that simply providing clear information does not ensure that good decisions will be made. Many factors not directly related to the actual information presented can affect decision-making. Irrational and/or emotional factors can be important determinants of patient decisions [27,28]. Misunderstanding or misinterpretation of even clearly presented information can contribute to poor decisions [29,30]. Presenting the same information in different ways can result in different decisions, suggesting that how the information is presented, as well as what is presented, is important [31-33]. Furthermore, psychological states such as feeling unsure or unprepared correlate with decision quality [34,35]. To facilitate high quality decision-making, information must be presented in a way that reduces the likelihood of misinterpretation, reduces uncertainty, and increases a feeling of being prepared for the decision [35,36].

Decision quality can be a difficult concept in situations where there is no objectively correct answer. The treatment decision literature [37,38] distinguishes between two kinds of decisions. Effective care decisions are those where clinical evidence suggests a course of action that has a benefit/harm ratio superior to all other available options. In such situations, a 'good' decision typically involves choosing the most effective option. In contrast, 'preference-sensitive' decisions have no clinically correct course of action, either because evidence on treatment effectiveness is unavailable, or because the benefits and harms of different treatments need to be evaluated in the context of patient values. It is for these preference-sensitive decisions where defining decision quality can be challenging. However, more than 20 years of work on the issue points to three critical components: a knowledge of the key aspects of the decision, accurate perceptions of the probabilities of outcomes under the different options, and a match between what outcomes patients value and the treatment options they choose [37,39].

The decision to participate in a clinical trial is an excellent example of a preference-sensitive decision. The pros and cons associated with participation (including, but not limited to, the benefits and harms of offered treatments) are frequently not well known; this is the reason for conducting the trial. As a result, decisions about whether to participate depend entirely on how individuals value the potential benefits (*e.g.*, incentives, potential health benefits, altruism) and harms (*e.g.*, side effects, clinic visits, travel) of participation. It is precisely for preference-sensitive decisions like these that patient decision aids (DAs) have been developed.

DAs are tools designed to help people make specific and deliberative choices among options by providing, at a minimum, information on the options and outcomes relevant to the person's health status. They can also include exercises to help people explicate choice predisposition, preference for role in decision-making, and how they value the different options [40]. DAs are intended to be used prior to, and in conjunction with, decision-making counselling sessions, and are thus consistent with the notion that consent should involve a process, not just a document.

The effectiveness of DAs has been tested extensively, with over sixty trials completed or in progress [40]. DAs have been shown to improve the quality of preference-sensitive patient decisions, in comparison to both standard care information documents and standard counselling strategies [35,36,39,41,42]. Specifically, they reduce uncertainty surrounding decisions (often termed decisional conflict [40,43]), enhance knowledge of key aspects of the decision and outcome probabilities [40,44,45], improve satisfaction with choices made, and improve the likelihood that selected treatments will be consistent with valued outcomes [44,46]. We propose that similar benefits might be attained when deciding whether to participate in a clinical trial. Furthermore, the related findings that DAs improve understanding, that improved understanding can increase trial participation rates [47-50], and that DAs can increase selection of underused treatment options [51,52] lead to the intriguing possibility that DAs may increase trial participation in situations where benefits compare favourably to harms.

Patient DAs have a strong theoretical foundation in the Ottawa Decision Support Framework [53,54], an evidence-based framework informed by cognitive, social, and organizational psychological theory, components of which have been validated in at least twelve studies [54]. This framework guided the development of the International Patient Decision Aid Standards (IPDAS) [36]. These standards were developed using an extensive evidence-based consensus process that included input from patients, practitioners, policy-makers, and decision support experts from fourteen countries worldwide. The IPDAS standards describe detailed recommendations about the content and delivery of information to facilitate high quality decisions. These standards are often consistent with, but sometimes more specific than, consent form guidelines. For example, while consent form guidelines require general information on benefits and harms of trial participation, IPDAS standards necessitate consistent denominators, time periods, and multiple (positive and negative) frames for outcome probabilities [36,55-57]. Furthermore, the IPDAS criteria also describe other exercises, such as requiring decision-makers to clarify which outcomes (positive and negative) they value most (*e.g.*, How important to you is an X% chance of improvement? How important is a Y% chance of a side effect?). Such exercises are commonly used in the patient DA literature, but rarely in the context of informed consent documents.

The decision support literature is increasingly focused on the development of computer-based (*i.e.*, 'online') decision aids. For information producers, the benefits of presenting DAs online include easy updating compared to print media, and easy dissemination via the internet [55]. For patients, advantages include accessibility and the potential for improved learning if multimedia tools are employed correctly [58]. Multimedia approaches as a class have met with limited success [36,48], but our preliminary research suggests that multimedia DAs can be effective when informed by a theoretical framework [59]. Therefore, the DAs developed for this study will be designed for presentation online.

To summarize, we propose that many failures of the existing informed consent process stem from an inappropriate focus on disclosure of information, rather than on facilitating high quality decision-making among potential research participants. In order for the informed consent process to allow both disclosure and understanding, innovative ways of presenting increasingly complex information to decision-makers are required. Patient DAs, which have been shown to improve decision-making in other contexts, may improve the quality of trial participation decisions. The current study will investigate this issue.

## Objectives

This study has four main objectives:

First, to examine whether consent forms of recently completed randomized controlled trials (RCTs) conform to standards for promoting high quality decision-making. Specifically, we hypothesize that there will be considerable variation in adherence to existing standards, even among a relatively homogeneous sample of consent forms drawn from investigator-initiated health research RCTs, and that many consent forms will lack key components necessary to facilitate high quality decision-making, as indicated by existing standards.

Second, to learn about the experience of trial recruitment from participants. Specifically, we will interview trial participants about: how they were recruited to participate in the trial; what factors they considered when deciding whether to participate; their impressions and reported use of any decision support materials provided; suggestions about how the recruitment process might have been improved; and overall impressions of trial participation.

Third, to employ a treatment DA template and user testing via the user-centered design (UCD) approach to develop a DA for people deciding whether to participate in a clinical trial. Specifically, we hypothesize that a template designed to inform development of patient DAs can be effectively used to develop a DA about whether to participate in a clinical trial, and that DA development via UCD can result in a DA that meets previously determined usability goals

Fourth, to test whether trial participation decisions based on a user-tested patient DA (as opposed to a standard consent form) will result in measurable differences in decision quality among hypothetical candidates for clinical trials. Specifically, we hypothesize that people using a DA will be less uncertain about the decision [60-63]; better remember the key aspects of the decision [45,64-71]; better understand probabilities of key outcomes [44,45,63,72-74]; show a higher correlation between outcomes valued and choice made [40,44,46]; and, be more likely to participate in the clinical trial [47,51,52,75].

## Methods

### Objective One: comparing consent forms to standards

Before developing a tool to help people decide whether to participate in a clinical trial, it will be important to investigate the effectiveness of the current process. Objective one will examine how well existing consent forms conform to empirically developed standards for promoting high quality decisions.

The primary tool for this assessment will be a checklist recently developed as part of the IPDAS [36]. Designed by an international collaboration of experts on patient decision-making, this checklist includes 74 criteria from 12 quality domains; each of these criterion are considered important for helping patients make difficult decisions about treatment or screening. The IPDAS criteria overlap with guidelines for informed consent documents (*e.g.*, use of plain language, reading level requirements, disclosure of conflicts of interest, presenting both positive and negative outcomes associated with the different options). As such, evaluating consent forms using this checklist will also assess requirements laid out in consent form guidelines. For completeness, consent form recommendations from other resources (*e.g.*, U.S. National Cancer Institute, National Cancer Institute of Canada, Tri-Council Policy Statement [76,77]) will be examined and any identified missing items will be appended to the checklist.

We will then assess a random sample of consent forms from approved investigator-initiated trials completed within the last six to 24 months at two institutions. The random sample of clinical trials will be drawn from local research ethics boards (REB) databases. Although these databases contain information on all institution-specific research projects, only non-industry studies labelled as clinical trials involving adults will be eligible for inclusion. Principal investigators of included studies will be contacted directly for consent forms and assured that identifying information (*e.g.*, investigator and proprietary drug names) will be removed before assessment. They will be informed that results will be reported in aggregate, meaning that individual studies will not be identified. Principal investigators will also be asked for information regarding overall enrolment rates; this should be known since the sample of consent forms will be limited to studies completed within the last six to 24 months. If consent forms for any of the target trials cannot be obtained, a replacement study will be randomly selected from the same review board database.

Study investigators who are approached to provide consent material for this study may feel pressured to comply because some of the authors are members of the local REBs to which they may later submit protocols. An analogous situation is common in clinical research, where physician-investigators recruit their patients into their own studies. In that situation, recruitment materials commonly include information designed to reassure patients that their care will not be affected by their decision to accept or decline trial participation. Similarly, we will reassure investigators that subsequent REB reviews will be unaffected by their decision to participate in our study. Furthermore, no investigator will review consent materials until all identifying information has been redacted from the documents. One investigator's name (RS) will be left off all Ottawa recruitment letters; it was felt his name may carry particular weight as he is the chair of the Ottawa REB. Furthermore, we will ensure that RS does not review any Ottawa consent materials, even after redaction.

A research coordinator and graduate student will be designated as coders and asked to rate all target consent forms with respect to the IPDAS checklist, using a Yes (2), Partly (1) and No (0) response scale for each criterion. For each consent form, the coders will also extract several descriptive factors that will later become the focus of *post hoc *exploratory analyses. For example, each study will be coded according to medical discipline (*e.g.*, oncology), and trial phase (*e.g.*, phase one, phase two). Exploratory analyses will then be used to look for correlations between consent form quality and these descriptive factors, as well as the relation between quality and true recruitment rate.

### Sample size and analyses

Consent forms will be randomly selected (25 from each institution) for application of the standards checklist. Assuming that compliance with 60% of the IPDAS items suggests a reasonable level of compliance, a sample of fifty consent forms allows for the detection of an overall compliance of 60% (30 of 50) with 95% confidence intervals of ± 15% [78]. This sample size will allow us to quantify the certainty of our estimates of the overall compliance with IPDAS criterion in the larger population of consent forms in the two databases.

Although the IPDAS checklist was developed according to a rigorous Delphi methodology, this document has not yet been validated as an assessment tool [36]. As a result, the investigator team will first 'pilot' the rating of several consent forms, thereby evaluating the checklist for overlapping, unclear, or missing items. These piloted consent forms will come from a database of publicly accessible consent documents already in the possession of the authors [79]. Once the items in the checklist have been agreed upon, the investigator team will train the two coders using these same pilot consent forms. This training will proceed until the consistency of coder agreement exceeds 80% on various components of the checklist. While coding the target consent forms, the two coders will resolve disagreements by consensus or confer with the investigator team when there is uncertainty. Inter-rater agreement for each item will be assessed using Kappa scores [80,81]. Because the checklist has not been validated overall as a scale of consent form quality, we will not compute overall assessment scores, but instead only examine descriptively the presence or absence of specific criteria.

Descriptive analyses will be used to evaluate the number and variation of checklist items present across the different consent forms (hypothesis one). Also, descriptive analyses will be used to identify which specific IPDAS components are more or less likely to be included in consent forms (hypothesis two). Further *post hoc *exploratory analyses will examine whether consent forms from oncology trials (an area where a significant work on consent form ethics has been conducted) include more components conducive to good decision-making than trials from other areas, and determine the relationship between consent form quality, as indicated by items on the IPDAS checklist, and true enrolment rates.

### Objective Two: interviews of trial participants

Objective one seeks to assess the current practice of trial recruitment by evaluating existing written materials. However, studying trial recruitment should not be limited to the written materials; other factors, such as consultation with study personnel, often play an important role in this process. Despite attempts to improve the informed consent process [48], relatively few studies have described the experiences of those individuals who must understand the complex information presented in consent documents; those that have focus on specific clinical areas [7]. Objective two will elicit the experiences of participants from a variety of studies, to identify themes that may be broadly applicable to improving the quality of participation decisions.

We will interview recent recruits from a convenience sample of ongoing clinical trials at local institutions. Our aim is not to document an exhaustive list of recruitment issues for each study, but rather to elicit themes that are common across trial recruitment situations. The authors will target eight to ten adult participants from multiple studies in five disciplines (oncology, thrombosis, emergency medicine, transfusion research, cardiology). Study investigators will contact the lead investigators of the selected RCTs and ask them to distribute recruitment letters to participating patients, if ethical circumstances allow. Our purposive sample will include both low- and high-risk studies (as determined by the local REB records) from each discipline, to elicit opinions about a range of studies.

Our phenomenological approach will involve semi-structured interviews approximately 45 minutes in length consisting of questions focused on trial recruitment, provided materials, decision-making, and how the overall process could have been improved. Three pilot interviews will be conducted to test the appropriateness and flow of the interview guide; the interview questions will be modified accordingly before proceeding with the remaining interviews. Participants will be prompted to provide clarification and elicit more detail, and all interviews will be recorded and transcribed. Participants will be offered $20 as a token of appreciation and to cover any attendant costs. Qualitative analysis will use NUD*IST software, applying the constant comparison method described by Strauss and Corbin [82] to elicit clusters of meanings from the narrative data that describe the experience of participants and inform the design of subsequent DAs.

### Objective Three: iterative development of a decision aid

Considerable work has examined how best to present complex information via computers [83-86]. Problems with online information can be characterized in terms of two dimensions: usability and usefulness [86]. Usability refers to the ease with which specified users can locate and interpret the information, while usefulness describes the degree to which the right information is presented at the right time. UCD is a qualitative, multi-stage procedure, and is one of the most well studied, efficient, and cost-effective methods for improving both the usability and the usefulness of complex, online materials [87]. It is an iterative process of design, evaluation, analysis, and re-design intended to create a final product that meets predetermined usability goals (*e.g.*, 90% of the time, patients should be able to read and complete the DA in less than 30 minutes, and score 80% or better on a knowledge test of the key aspects of the decision). This process has been shown in a variety of contexts to improve user satisfaction [88], reduce errors in navigation and the resulting confusion [87,89], and to increase the efficiency with which the information can be found [86]. However, this technique has not yet been applied to decision support materials for people making health care decisions.

We propose to employ UCD as a qualitative methodology designed to optimize the IPDAS DA template for decisions involving participation in clinical trials. This template was developed for screening and treatment decisions, where the benefits of using DAs have been clearly demonstrated. However, neither the template nor the generalized DA technology has been tested in the context of clinical trial participation. As a result, some detailed, qualitative pilot testing is required to examine how a DA based on the IPDAS template mediates decision making in this context.

We will develop DAs for two target studies from the set of trials assessed in objective one. Although UCD testing is labour intensive, developing two DAs instead of one will help identify which issues can be generalized and which issues are idiosyncratic to specific studies. The choice of which two trials to focus on will be determined by two main criteria. First, studies whose inclusion criteria are extremely strict, or where the relevant population does not exist locally, will be avoided to allow enough participants to be recruited for objective four. Second, if there is significant variation in the extent to which consent forms adhere to standards (objective one, hypothesis one), the investigator group will choose one trial that meets relatively few criteria and one that meets more. This selection process will allow us in objective four to study whether a DA only affords a benefit over poorly designed consent forms. Note that analysis for this objective will be exploratory in nature, since any differences in the number of criteria met will be confounded with clinical condition.

Risk information will be consistent for both consent forms and DAs (*i.e.*, the DA will not introduce any new risk information). While both the DA literature and the IPDAS criteria recommend providing specific numbers associated with the risks of different outcomes, such specific outcome probabilities are often not available for clinical trials. Therefore, for the purposes of this project, specific outcome probabilities will not be included in the DA if they are not provided in the associated consent form. Instead, the DA will contain standardized descriptors, such as those recommended by the National Cancer Institute of Canada (*e.g.*, common = > 200 per 1000, very rare =< 1 per 1000) [90].

Data collection for this objective will consist of two phases of qualitative UCD testing: expert testing and user testing. Phase one will involve experts (three DA experts and three content areas, drawn from the investigator team and colleagues) working through the DA to ensure that all information relevant to the decision is present. They will examine the DA to ensure formatting conforms to basic principles or 'heuristics' of good design (heuristic evaluation [91]). These experts will also identify potential stumbling blocks in the material by working through the entire tool; this technique is referred to as a 'cognitive walkthrough' [92].

Once the expert evaluations are complete, phase two will subject the updated version of the DA to a series of 'user tests' involving adult participants 'talking aloud' [93] as they work through the tool. The user tests will be videotaped and evaluated for user misunderstandings, expressions of frustration or confusion, and the specific areas of the DA where these occurred. These 'usability problems', as well as items that multiple users identify as challenging, will become target areas for improvements on subsequent iterations. The DA will be revised after each iteration of five or six participants [86]. This iterative approach provides (in the first iteration) baseline measures of user satisfaction and performance (time required to read, comprehension, misunderstandings), as well as (in later iterations) the degree to which the current version of a DA meets pre-specified usability goals. Each session will take approximately 45 to 60 minutes, for which participants will be offered $20 as a token of appreciation and to cover any attendant costs.

### Sample size and analyses

Participants in this phase of the study will be naïve volunteers age-matched to typical patients with the condition discussed in the DA. Based on previous experience and the usability testing literature [93], four to five iterations of five to six participants each will be sufficient to meet the usability goals described above (*i.e.*, twenty to thirty participants will be required).

### Objective Four: prospective observational study

Objective four will compare the experiences of people using consent forms and DAs to assist hypothetical decisions about trial participation. This objective will consist of a prospective observational study designed to collect both qualitative and quantitative data relevant to whether this approach warrants further evaluation with a pilot RCT.

Participants will be naïve individuals who meet the inclusion criteria of the target study, and thus could have been approached to participate in the original study. However, those who actually were approached to participate in the target study, regardless of their decision to participate, will be excluded from our study. In addition to the type of decision tool (consent form, DA), the two consent forms that were subjected to user testing from objective three will be the focus of this study. Participants will be eligible for our study if they speak English, are over 18 years of age, and meet the inclusion criteria of one of the target studies.

Potential participants that meet one of the two sets of inclusion criteria will be approached to enrol in our study (*i.e.*, non-random allocation to target study), and standard consent will be obtained. Participants will work through one of the two decision support tools. Data will first be collected on consent forms, then later for DAs. We have chosen this approach for two reasons. First, by collecting data for consent forms initially, we will not need to wait until the end of DA user testing to begin data collection for objective four. Participants may need to fit strict inclusion criteria for the relevant studies chosen for objective four; this approach adds flexibility to our timeline. Second, we will incorporate information gleaned from the consent form participants (particularly their qualitative responses) to further improve the DA. This approach sacrifices the experimental rigour of an RCT, but adds a richness of qualitative and quantitative data that is most likely to result in both a tool that maximally improves the informed consent process, and in a better understanding of what outcomes are affected by the newer decision support tool.

After working through the decision support tool, participants will complete a paper-based questionnaire. This questionnaire will include validated measures of constructs related to decision quality, as well as qualitative questions about their impressions of recruitment process and materials. Quantitative outcomes measured will include decisional conflict [94], memory for key aspects of the decision, knowledge of the probabilities of different outcomes, values associated with different outcomes for comparison with the participation choice, and participation choice. We will also measure satisfaction with the decision support materials, satisfaction with the informed consent process [49], and anticipated regret of key negative outcomes. In addition, participants will be asked to make a hypothetical decision about whether or not they would participate in the target trial (yes, no, unsure). Of note, participants will not have access to the decision support materials when completing the questionnaire. At the end of the session, participants will be provided with a debriefing form, explaining the purpose of the study and how their data will contribute to towards improving the consent process. The entire session will take 45 to 60 minutes, for which participants will be offered $20 as a token of appreciation and to cover any attendant costs.

### Sample size and analyses

The sample size calculation was based on detecting differences on the continuous decisional conflict scale [43]. The authors selected this scale as the primary outcome for this analysis because it is considered a key correlate of good decision quality and has been well validated in the context of many treatment decisions. Sample size calculations were carried out by simulation using the AOV function of R statistical software [95]. We conducted a simulation with 50,000 iterations, detecting a 10% difference on a continuous outcome. Results of the simulation showed that a sample size of 30 individuals per group, or 120 in total, yields a proportion of rejecting the null when it is true of 0.048 (*i.e.*, alpha level is approximately 0.05), while the proportion of incorrectly accepting the null hypothesis is less than 0.01 (*i.e.*, power is greater than 0.99).

Analyses for this study will consist of linear (for continuous outcomes) and logistic regressions, with type of decision support (consent form/DA), target study, and their interaction predicting the different outcomes. For example, when predicting decisional conflict, a significant effect of type of decision support will indicate whether those making decisions on the basis of consent form and DA differ in terms of how unsure they remain about the decision. Similarly, a significant effect of target study will demonstrate whether satisfaction was higher for one condition regardless of type of decision support, and their interaction will show whether the two effects are independent. The collected demographic characteristics of respondents (*e.g.*, age, sex) will also be included as covariates.

Hypotheses for this objective were principally derived from literature on the effects of DAs on treatment decisions, *i.e.*, that they improve the quality of decision-making. The authors hypothesize that using a DA will result in: reduced indecision and decisional conflict [39,40,45,61,62]; improved memory for key aspects of the decision [64,96]; improved knowledge of key outcome probabilities [40]; and a higher correlation between self-identified important outcomes and the selected treatment choice [40,97]. Literature has shown that DAs can affect behavioural outcomes, such as increased use of underused treatments [51,52], and that that confusion arising from consent forms may contribute to non-participation [48]. As a result, we further hypothesize that DAs may increase participation in trials where risk/benefit ratios are favourable.

Finally, we will collect and analyze the number and content of questions that potential participants ask after working through the decision support materials. These post-consent form discussions will not only serve to make the consent process more representative of real world recommended practice [48], they will also serve as a valuable data collection opportunity. The DA will explicitly ask people to record any unanswered questions about the associated trial, while encouraging systematic thinking about the various possible outcomes. As a result, we expect that more questions, and more detailed questions, will stem from those working through the DA as opposed to the consent form. The enrolment rates of the sample consent participants will be compared to the reported trial enrolment rates, to estimate how closely hypothetical recruitment mimics real life situations.

## Limitations

A number of limitations of this study warrant consideration. First, the development of a DA to better inform trial participation decisions does not address all the ethical concerns related to informed consent. It has also been argued that the existing informed consent process lends itself to problems by focusing on specific, isolated decisions, rather than larger concepts such as overall autonomy of the individual (*e.g.*, see Kukla [98]). While the current approach does not directly address these larger issues, we believe that the development of improved decision tools will serve such larger goals. For example, improved decision tools will encourage thinking about informed consent as a process rather than a discrete event. Furthermore, DAs may elicit benefits beyond the immediate aims of this study by explicitly addressing issues such as the balance between benefits and harms, and prompting potential participants to think about what further information they require and their preferred role in decision-making [7]. Since memory for information presented during the consent process can fade throughout participation [49], the DAs developed for this study will include a take home one-page summary that can be used to periodically review key trial information. Future work, perhaps involving a larger study examining the entire time course of trial participation, will be required to consider these larger ethical concerns.

Second, the IPDAS checklist used in objective one has not been validated as an assessment tool. This checklist was developed according to a modified Delphi method [99], and constitutes the consensus of an international consortium of experts on which items comprise high quality decision support. Because the checklist has not been formally validated, we decided to incorporate a pilot testing phase designed to identify overlapping or problematic items and describe item-specific results; an overall 'quality' score will not be computed. Future work should involve formal psychometric analysis of the IPDAS checklist as a measure of DA quality in treatment decisions, and separately as an indicator of the ability to improve informed consent.

Third, objectives three and four will make use of hypothetical decision-makers rather than actual patients making real world decisions. This characteristic is common in the literature, but has been argued to adversely affect study generalizability [48]. However, increasing evidence shows that decision-making based on hypothetical, written scenarios is highly correlated with real world decisions [100,101]. This study is designed to determine whether incorporating DAs into real informed consent decisions is worthwhile; as such, we felt that it would be inappropriate to use actual patients until it is known whether DAs are at least as effective as standard practice in assisting decision-making. However, it may be that in this context, hypothetical decisions are not predictive of actual decisions. This issue will be addressed by examining the calibration of hypothetical enrolment rates from objective four with true enrolment rates collected in objective one. Determining the usefulness of DAs for true participation decisions will be the subject of another study.

Fourth, objective four will compare online DAs to existing, paper-based consent forms, which are still the current norm for most clinical trials. This comparison leaves open the possibility that any observed variation could stem from the difference in media (paper-based versus online) rather than differences in the decision support tool itself (consent form versus DA). However, a recent systematic review of interventions designed to improve informed consent documents showed that presenting the information online is not enough to ensure better understanding, and a meta analysis of all multimedia manipulations showed a null effect on consent form knowledge [48]. As a result, we expect that any response differences between the two types of decision material will be primarily due to variations in the support framework, rather than any implicit advantage in the display medium.

Fifth, blinding, allocation concealment and randomization for objective four are impossible or impractical, and thus strong claims about the relative benefits of DAs versus consent forms cannot be made. The next step in this research program will address this issue by comparing the performance of DAs and consent forms in the context of a real world RCT.

Sixth, this work will focus only on investigator-initiated trials, and not industry-sponsored trials. Practical and legal aspects of studying industry trials led us to limit our samples for this project; however, this subgroup of trials is clearly of interest and will be the subject of a separate investigation.

Finally, examination of long term effects of the informed consent process, such as dropout rates, satisfaction or regret with participation in the trial, willingness to participate in a similar trial, etc., will not be possible in this short term project, and will be the subject of future work. The current study will examine only immediate outcomes that in the treatment decision literature are known to be correlated with the longer term outcomes [102].

## Competing interests

The authors declare that they have no competing interests.

## Authors' contributions

JCB conceived the general research questions and wrote the proposal. AL, DAF, KGS, RS, and JK provided specific clinical and/or methodological expertise. AL and JCB wrote the study protocol. All authors contributed to the development of the specific research questions, reviewed the proposal and protocol, and read and approved the final manuscript.
